# Glue versus tackers for mesh fixation in laparoscopic inguinal hernia repair: a meta-analysis and trial sequential analysis

**DOI:** 10.1007/s10029-025-03315-w

**Published:** 2025-04-05

**Authors:** Samuel Kitching, Agastya Patel, Jacob Tan, Jacob Kadamapuzah, Thomas Satyadas

**Affiliations:** 1 Faculty of Biology, Medicine and Health, University of Manchester, Oxford Road, Manchester, UK; 2 Regional Hepato-Pancreato-Biliary Surgical Unit, Manchester Royal Infirmary, Manchester, UK; 3 First Doctoral School, Medical University of Gdansk, Gdansk, Poland

**Keywords:** Laparoscopic, Hernia, Mesh, Tackers, Glue, Fibrin

## Abstract

**Purpose:**

Mesh fixation in laparoscopic inguinal hernia repair has improved patient outcomes compared to natural tissue repair. The method of fixation of the mesh to the abdominal wall and its impact on patient outcomes has not been determined as part of a trial sequential analysis. The aim of this study is to compare the use of glue and tackers in mesh fixation of inguinal and femoral hernia repair by meta-analysis and trial sequential analysis (TSA).

**Method:**

Medline, Cochrane Library, Scopus, Web of Science, and EMBASE were searched to retrieve relevant randomised controlled trials (RCT) comparing glue and tacker fixation in laparoscopic inguinal and femoral hernia repair, resulting in 648 studies, of which 18 met the inclusion criteria. This data was systematically analysed using RevMan and TSA software.

**Results:**

2312 patients were included in the 18 RCTs used in this study, with 1149 in the glue cohort and 1163 in the tacker cohort. Glue fixation significantly reduced risk of haematoma formation [MD (95% CI): 0.35 (0.17–0.73), P < 0.01]. Glue fixation resulted in significantly less acute pain [MD (95% CI): − 1.80 (− 2.71 to − 0.89), P < 0.01] and chronic pain [MD (95% CI): 0.42 (0.27–0.64), P < 0.01]. Glue fixation also allowed significantly quicker return to normal activity/work compared to tacker fixation [MD (95% CI): − 1.92 (− 3.17 to − 0.67), P < 0.01]. TSA confirmed that glue fixation significantly reduced early pain scores (< 3 months) and haematoma incidence compared to tacker fixation.

**Conclusion:**

Mesh fixation with glue is superior to tackers in reducing post-operative pain and haematomas, which means patients return to work/activity significantly faster. Surgeons should be aware of these benefits when consenting the patient for laparoscopic inguinal and femoral hernia repair.

## Introduction

In laparoscopic inguinal hernia repair, glue or tackers are being used for the fixation of the mesh [1]. It is widely accepted that the laparoscopic approach to inguinal hernia repair provides better outcomes in terms of chronic pain compared to open hernia repair. [2, 3]. However, 5–6% of patients continue to experience chronic post-operative pain after inguinal hernia repair, but the effect of different fixation methods (glue versus tackers) on surgical outcomes is debated [4].

Fibrin glue was initially used in 1998 and is being increasingly used as a non-invasive approach, thought to increase wound healing via biodegradability, haemostatic properties and flexibility in the adhesive [5], distributing stress across the surface of the tissue compared to tackers which insert into the tissue in which they are placed. In addition, the application of fibrin glue avoids risk to neurovascular supply to the surrounding tissue in opposition to tackers.

Fixation of mesh is associated with postoperative pain caused by irritation of the surrounding nerves, bone and peritoneum [6], the difference in pain between fixation with glue or tackers has been explored in the literature by randomised control trial but no trial sequential analysis has been completed on all post-operative outcomes [7–20]. This study aims to perform a trial-sequential analysis of randomised controlled trials comparing postoperative outcomes following glue versus tacker mesh fixation.

## Methods

This study was designed according to an agreed protocol, which complied with Preferred Reporting Items for Systematic Reviews and Meta-Analyses (PRISMA) statement standards [21]. The protocol for the study detailing the research question, search strategy, criteria for inclusion and risk of bias assessment was established a priori.

### Eligibility criteria

Randomised controlled trials (RCTs), including patients undergoing laparoscopic inguinal hernia repair with glue and tacker mesh fixation, were considered eligible for inclusion. The intervention of interest was mesh fixation with glue. The comparison of interest was mesh fixation with tackers. The primary outcomes were acute and chronic postoperative pain and recurrence. Secondary outcomes were rates of postoperative complications (seroma, hematoma, surgical site infection), operative time, return to work/activity, and length of hospital stay.

### Search strategy and selection process

**Fig. 1:**
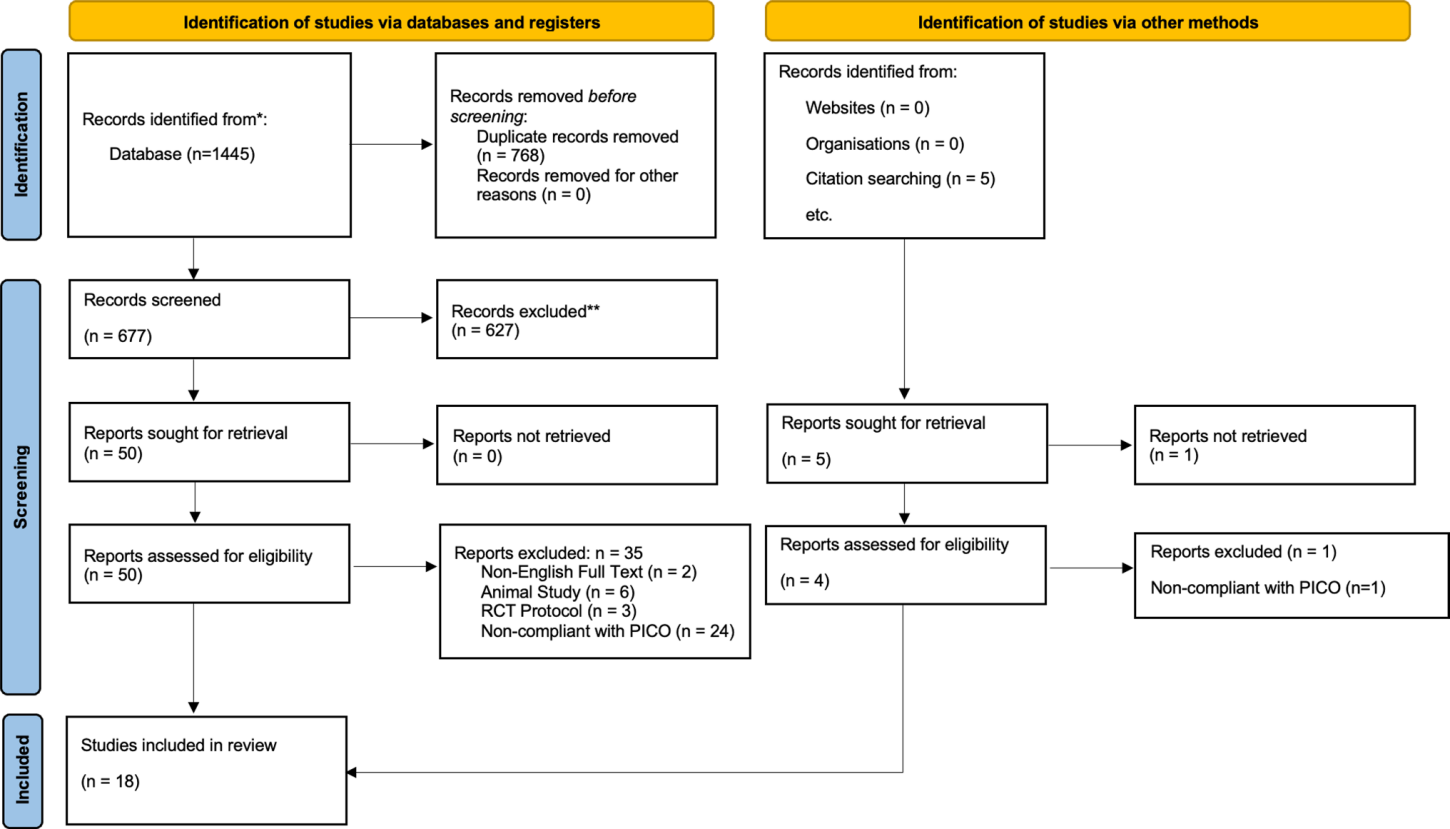
PRISMA flowchart

A comprehensive literature search of Embase, MEDLINE, Web of Science, Scopus, and the Cochrane Central Register of Controlled Trials (CENTRAL) databases was performed from inception to April 2024 to identify full-text articles related to the research question. The following search strategy, without any filters, was utilised: ("laparoscopic" OR "transabdominal preperitoneal repair" OR "TAPP" OR "total extraperitoneal repair" OR "TEP" OR "hernioplasty" OR "mesh fixation") AND ("glue" OR "fibrin" OR "tissue adhesive") AND ("staple*" OR "tack*"). The PRISMA flowchart describing the results of the search and screening process is provided in Fig. 1. The initial search process provided 677 articles, which were screened based on titles and abstracts by two independent authors (SK, AP). Additionally, five articles were identified via backward citation searching of the included studies. The full text of 50 eligible articles was reviewed against pre-specified inclusion criteria for the meta-analysis. Any disagreements between the reviewers were resolved through discussion to consensus (Table 1).

**Table 1:** Baseline patient characteristics, surgical intervention and intraoperative details of included studies

Author	Grouping	N patients	Male%	Age (years)	Hernia size	Laparoscopic technique	Operative time	Surgeons
Issa et al. 2021	Glue	46	96	48.5 (± 14.0)	NR	TEP	NR	13
	Tackers	54	96	57.9 (± 15.2)	NR		NR	16
Lovisetto et al. 2007	Glue	99	89	52.9 (± 14.6)	NR	NR	53.6 (± 7.6)	4
	Tackers	98	87	53.2 (± 12.6)	NR		39.6 (± 7.6)	4
Tolver et al. 2013	Glue	50	100	NR	NR	TAPP	39.3 (± 38.2)	2
	Tackers	50	100	NR	NR		51.3 (± 78.6)	2
Brügger et al. 2003	Glue	40	100	NR	NR	TAPP	105.0 (± 103.8)	NR
	Tackers	40	98	NR	NR		106.7 (± 96.1)	NR
Ambore et al. 2017	Glue	30	NR	NR	2.8 (± 1.0)	NR	NR	> 1
	Tackers	30	NR	NR	3.2 (± 0.7)		NR	> 1
Nizam et al. 2021	Glue	30	90	NR	NR	TEP	90.1 (± 5.6)	> 1
	Tackers	30	90	NR	NR		88.3 (± 5.1)	> 1
Fortelny et al. 2012	Glue	44	100	45.5 (± 11.3)	NR	TAPP	70.0 (± 19.0)	1
	Tackers	45	100	45.0 (± 14.0)	NR		69.0 (± 23.0)	1
Lau et al. 2005	Glue	46	98	NR	NR	TEP	75.2 (± 18)	> 1
	Tackers	47	100	NR	NR		76.7 (± 19.1)	> 1
Bunkar et al. 2021	Glue	30	NR	49.6 (± 16.9)	NR	TEP	NR	1
	Tackers	30	NR	48.8 (± 13.2)	NR		NR	1
Melissa et al. 2014	Glue	64	NR	52.8 (± 10.3)	1.6 (± 0.6)	TEP	75.8 (± 19.0)	2
	Tackers	65	NR	53.3 (± 11.8)	1.5 (± 0.6)		73 (± 21.3)	2
Boldo et al. 2008	Glue	22	NR	NR	NR	TAPP	NR	1
	Tackers	20	NR	NR	NR		NR	1
Olmi et al. 2007	Glue	150	98	NR	NR	TAPP	53.3 (± 29.9)	2
	Tackers	150	97	NR	NR		51.7 (± 33.7)	2
Chandra et al. 2015	Glue	50	76	40.6 (± 8.4)	3.0 (± 0.7)	TEP	50.3 (± 4.1)	> 1
	Tackers	50	70	41.7 (± 8.5)	3.2 (± 0.8)		54.9 (± 5.8)	> 2
Azevedo et al. 2022	Glue	21	100	NR	NR	TAPP	NR	> 1
	Tackers	21	100	NR	NR		NR	> 1
Habeeb et al. 2020	Glue	266	97	NR	NR	TAPP	NR	> 1
	Tackers	266	95	NR	NR		NR	> 1
Manish et al. 2023	Glue	27	19	NR	NR	TAPP	71.0 (± 3.2)	1
	Tackers	27	22	NR	NR		70.0 (± 3.0)	1
Liew et al. 2017	Glue	32	NR	52.2 (± 19.0)	NR	TEP	70.0 (± 24.0)	4
	Tackers	34	NR	57.0 (± 17.0)	NR		65.0 (± 15.0)	4
Jeroukhimov et al. 2023	Glue	102	94	54.5 (± 16.3)	NR	TEP	NR	5
	Tackers	106	93	54.5 (± 16.0)	NR		NR	5

*NR* not reported or in the correct format, *TEP* total extraperitoneal repair, *TAPP* transabdominal peritoneal repair

A structured data collection form was used by two independent reviewers (SK, AP) to extract data from included studies. Data were retrieved regarding baseline study and patient characteristics, surgical and repair technique (Glue or Tackers), intraoperative outcomes, and postoperative outcomes (Table 2).

**Table 2:** Data on postoperative outcomes from the included studies

Author		Grouping	VAS < 3 months	VAS > 3 months	Surgical site infection	Seroma	Haematoma	Recurrence	Return to work/activity	Stay in hospital	Chronic pain
Issa et al. 2021	Glue		3.6 (± 3.2)	0.8 (± 1.9)	NR	0	4	0	NR	NR	1
	Tackers		4.1 (± 3.0)	0.6 (± 1.7)	NR	0		0	NR	NR	0
Lovisetto et al. 2007	Glue		19.3 (± 5.3)	NR	0	0	3	1	7.9 (± 1.3)	NR	1
	Tackers		26.0 (± 5.3)	NR	0	0		0	9.1 (± 2.0)	NR	5
Tolver et al. 2013	Glue		34.3 (± 61.1)	NR	NR	NR	NR	2	NR	NR	NR
	Tackers		45.7 (± 74.8)	NR	NR	NR		0	NR	NR	NR
Brügger et al. 2003	Glue		4.7 (± 3.1)	5.3 (± 3.1)	0	0	3	2	19.7 (± 23.1)	5.3 (± 5.4)	NR
	Tackers		5.3 (± 3.8)	5.3 (± 3.8)	0	0		1	28.0 (± 37.7)	5.3 (± 5.4)	NR
Ambore et al. 2017	Glue		1.0 (± 0.5)	0	NR	0	0	0	1.0 (± 0.6)	2.0 (± 1.6)	NR
	Tackers		2.2 (± 1.4)	0	NR	1		0	3.0 (± 0.6)	3.0 (± 1.6)	NR
Nizam et al. 2021	Glue		4.7 (± 1.4)	1.5 (± 1.2)	NR	3	0	0	NR	49.3 (± 4.6)	NR
	Tackers		7.5 (± 1.5)	3.3 (± 1.8)	NR	10		0	NR	57.1 (± 6.0)	NR
Fortelny et al. 2012	Glue		2.1 (± 3.8)	2.3 (± 4.6)	NR	NR	NR	1	NR	4.5 (± 0.8)	NR
	Tackers		2.1 (± 3.8)	1.5 (± 2.7)	NR	NR		1	NR	4.2 (± 0.9)	NR
Lau et al. 2005	Glue		3.7 (± 2.3)	NR	0	16	0	0	3.3 (± 2.3)	1.0 (± 0.0)	5
	Tackers		3.7 (± 2.3)	NR	0	5		0	3.0 (± 1.5)	1.3 (± 0.8)	8
Bunkar et al. 2021	Glue		1.9 (± 1.1)	0	1	1	0	0	NR	NR	0
	Tackers		2.5 (± 1.6)	0.1 (± 0.5)	0	1		0	NR	NR	1
Melissa et al. 2014	Glue		NR	NR	5	11	0	0	NR	NR	3
	Tackers		NR	NR	2	7		0	NR	NR	1
Boldo et al. 2008	Glue		1.6 (± 2.4)	NR	NR	9	0	3	NR	NR	NR
	Tackers		5.0 (± 2.8)	NR	NR	8		2	NR	NR	NR
Olmi et al. 2007	Glue		NR	NR	NR	5	0	0	5.3 (± 3.7)	1.7 (± 1.5)	0
	Tackers		NR	NR	NR	12		0	11.3 (± 11.2)	1.7 (± 1.5)	3
Chandra et al. 2015	Glue		2.3 (± 1.0)	5.4 (± 1.6)	NR	10	0	0	NR	2.1 (± 0.4)	NR
	Tackers		5.8 (± 1.4)	2.1 (± 0.6)	NR	31		4	NR	2.4 (± 0.6)	NR
Azevedo et al. 2022	Glue		0	0	NR	1	NR	0	NR	NR	0
	Tackers		0	0	NR	2	NR	0	NR	NR	0
Habeeb et al. 2020	Glue		NR	NR	0	NR	NR	2	NR	NR	19
	Tackers		NR	NR	1	NR	NR	1	NR	NR	53
Manish et al. 2023	Glue		1.5 (± 0.5)	0	NR	1	0	NR	NR	NR	0
	Tackers		2.2 (± 0.7)	0	NR	1	4	NR	NR	NR	0
Liew et al. 2017	Glue		NR	NR	NR	7	1	0	NR	NR	2
	Tackers		NR	NR	NR	8	0	0	NR	NR	0
Jeroukhimov et al. 2023	Glue		NR	NR	NR	NR	0	1	NR	NR	NR
	Tackers		NR	NR	NR	NR	1	9	NR	NR	NR

*NR* not reported or in the correct format, *VAS* visual analogue score

### Risk of bias assessment

The Cochrane Risk of Bias 2.0 (RoB 2.0) Tool was used to assess the risk of bias within included RCT studies. Two authors (SK, AP) carried out the risk of bias assessment independently, with any disagreements resolved by consensus.

### Statistical analysis

Pooled summary estimates for continuous variables were performed using mean difference (MD) and for categorical variables using odds ratio (OR) with respective 95% confidence interval (CI). All statistical analyses were modelled based on a 95% confidence level to demonstrate statistical significance. If studies reported continuous variables as median (interquartile range), Wang et al.’s method was utilised for conversion into estimated mean ± standard deviation and used in the statistical analysis [22]. Heterogeneity between studies was measured using the I^2^ statistic, with I^2^ > 50% considered to indicate significant statistical heterogeneity. Summary estimates were produced using a random-effects model (in case of significant heterogeneity) or a fixed-effects model. Forest plots were generated for visual representation of analysed outcomes to assess publication bias. The Review Manager (RevMan, version 5.0. Copenhagen, 2014) software was used for data synthesis.

Trial sequential analysis (TSA) software (0.9.5.5 Beta, Copenhagen Trial Unit, Denmark) was used to perform trial sequential analysis. TSA of data from randomised controlled trials was conducted when an outcome was reported by at least two randomised trials. To assess the likelihood of type 1 error, the O’Brien-Fleming α-spending function was used to adjust the thresholds for the Z-values. Furthermore, the Z values were penalised using the iterated logarithm law. To assess the likelihood of type 2 error, the β-spending function and futility boundaries were used. Random effects models were used for TSA, and constant continuity correction was used to deal with the no-event RCTs.

## Results

**Fig. 2:**
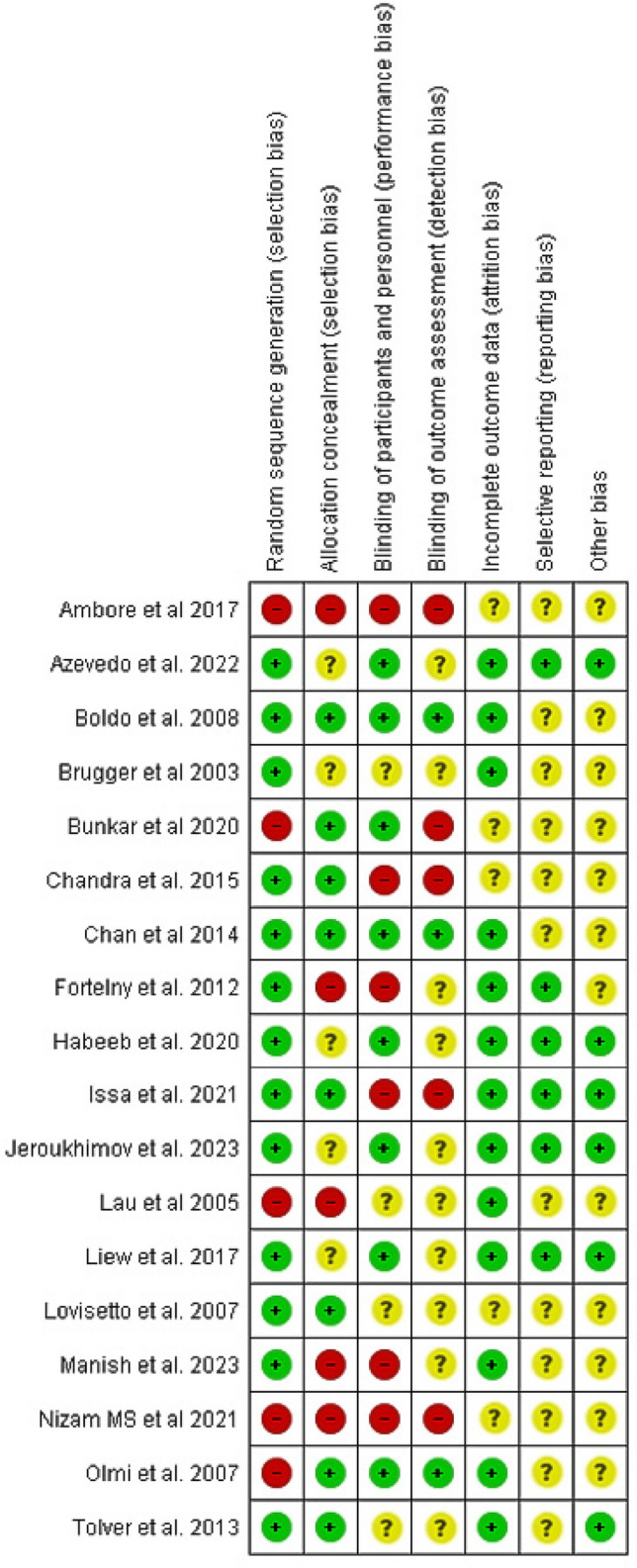
Risk of bias assessment

In total, 18 RCTs were eligible for inclusion in the meta-analysis (Fig. 1). Overall, 2312 patients were included in this study, of whom 1149 underwent glue fixation and 1163 underwent tacker fixation. Figure 2 highlights the outcomes of the methodological quality assessment based on the Cochrane tool.

Both groups included participants with a hernia of less than 5 cm, with a mean size of 2.46 cm and 2.63 cm for Glue and Tacker groups, respectively. The included studies only treated inguinal hernias, with three studies treating femoral hernias in addition. Eight studies performed totally extraperitoneal repair (TEP) [7, 13, 14, 18, 19, 23–25], eight studies did transabdominal pre-peritoneal repair (TAPP) [9, 10, 12, 16, 17, 26–28], and two studies did not describe the technique used [8, 11]. Four studies used data from a single surgeon [12, 14, 16, 27], 13 studies reported data from more than one surgeon [7, 8, 11, 13, 17–19, 23–26, 28, 29], and one did not report the number of surgeons involved in the study [10].

### Intraoperative outcomes

**Fig. 3:**
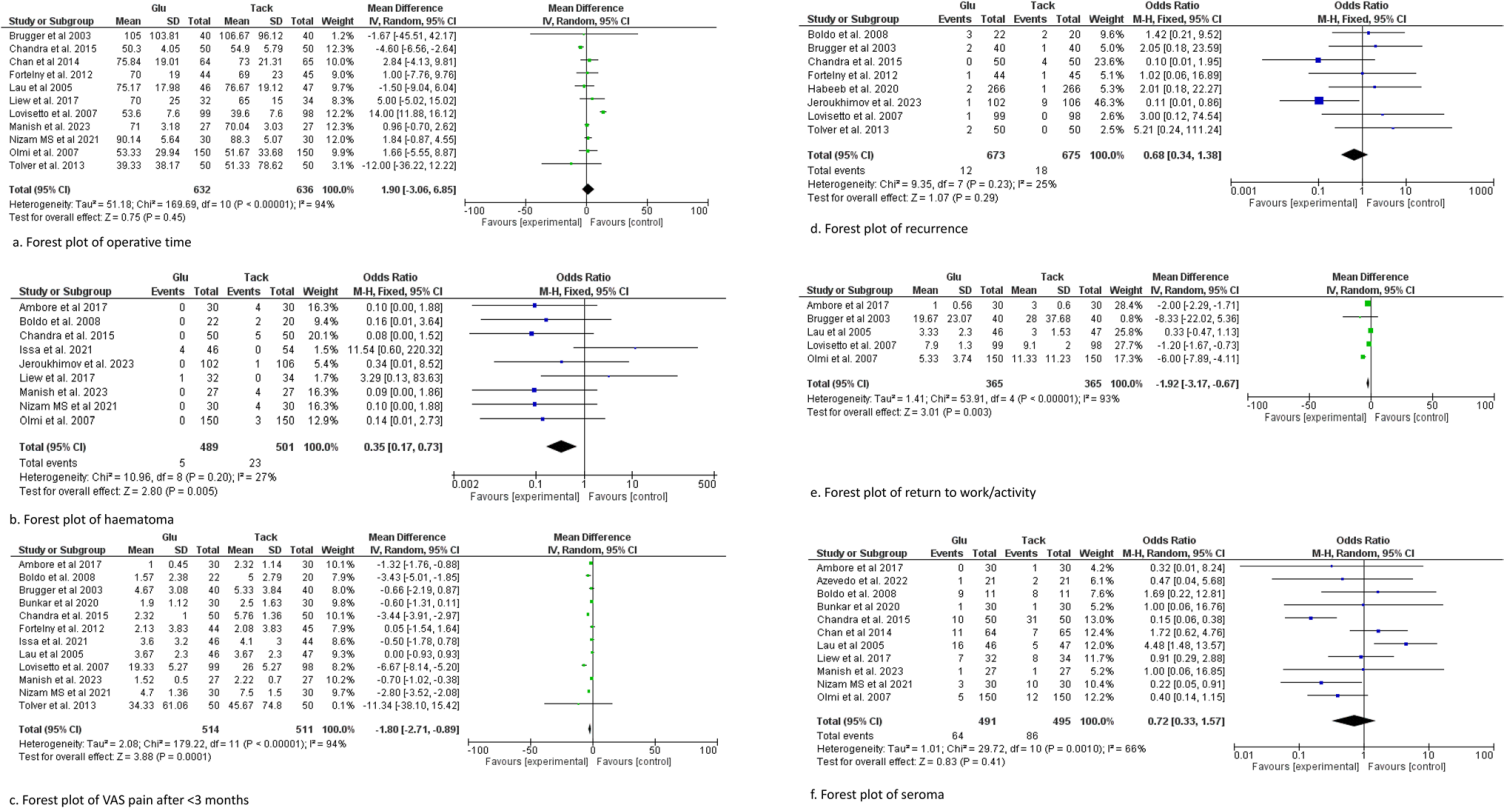
Forest plot of operative time (**a**), haematoma (**b**), VAS pain after < 3 months (**c**), recurrence (**d**), return to work/activity (**e**) and seroma (**f**)

The only intraoperative outcome included in the trial sequential analysis was operative time, with 11 studies reporting operative time with an average operative time of 68.52 ± 24.76 min and 67.90 ± 28.03 min for the glue and tacker cohort, respectively (n = 1268) [8–10, 12, 13, 15, 17–19, 25, 27]. The utilization of glue compared to tacker fixation showed no significant difference in operative time [MD (95% CI): 1.90 (− 3.06 to 6.85), P = 0.45, I^2^ = 94%] (Fig. 3a).

**Fig. 4:**
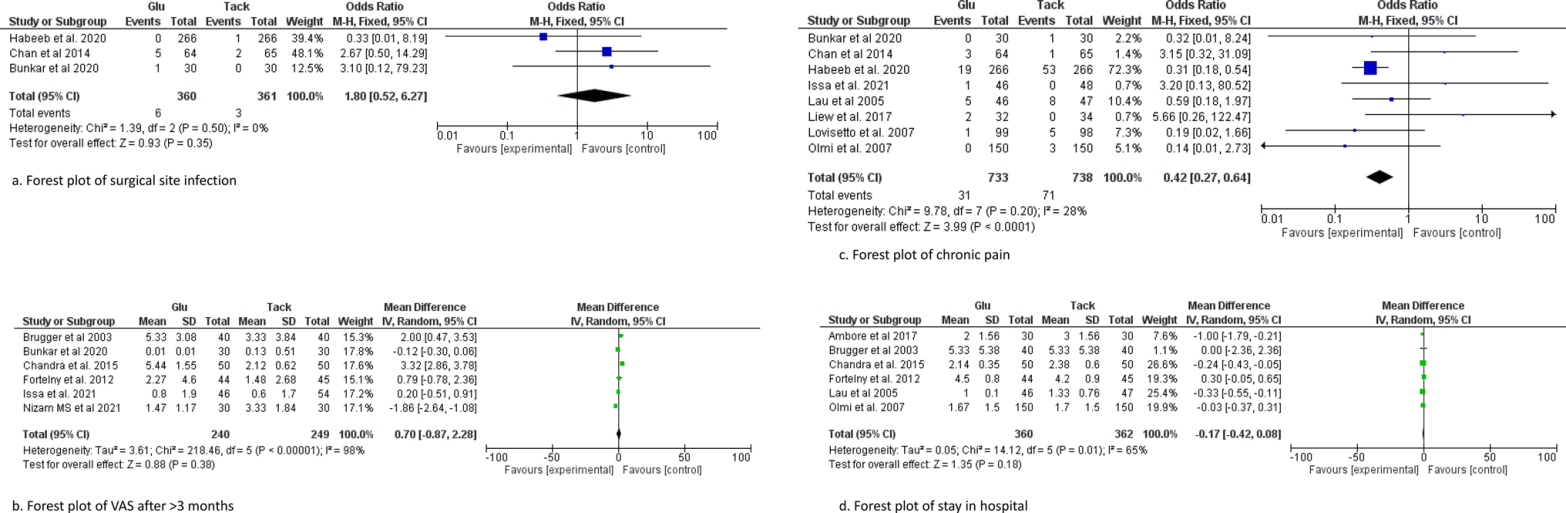
Forest plot of surgical site infection (**a**), VAS pain after > 3 months (**b**), chronic pain (**c**), stay in hospital (**d**)

**Fig. 5:**
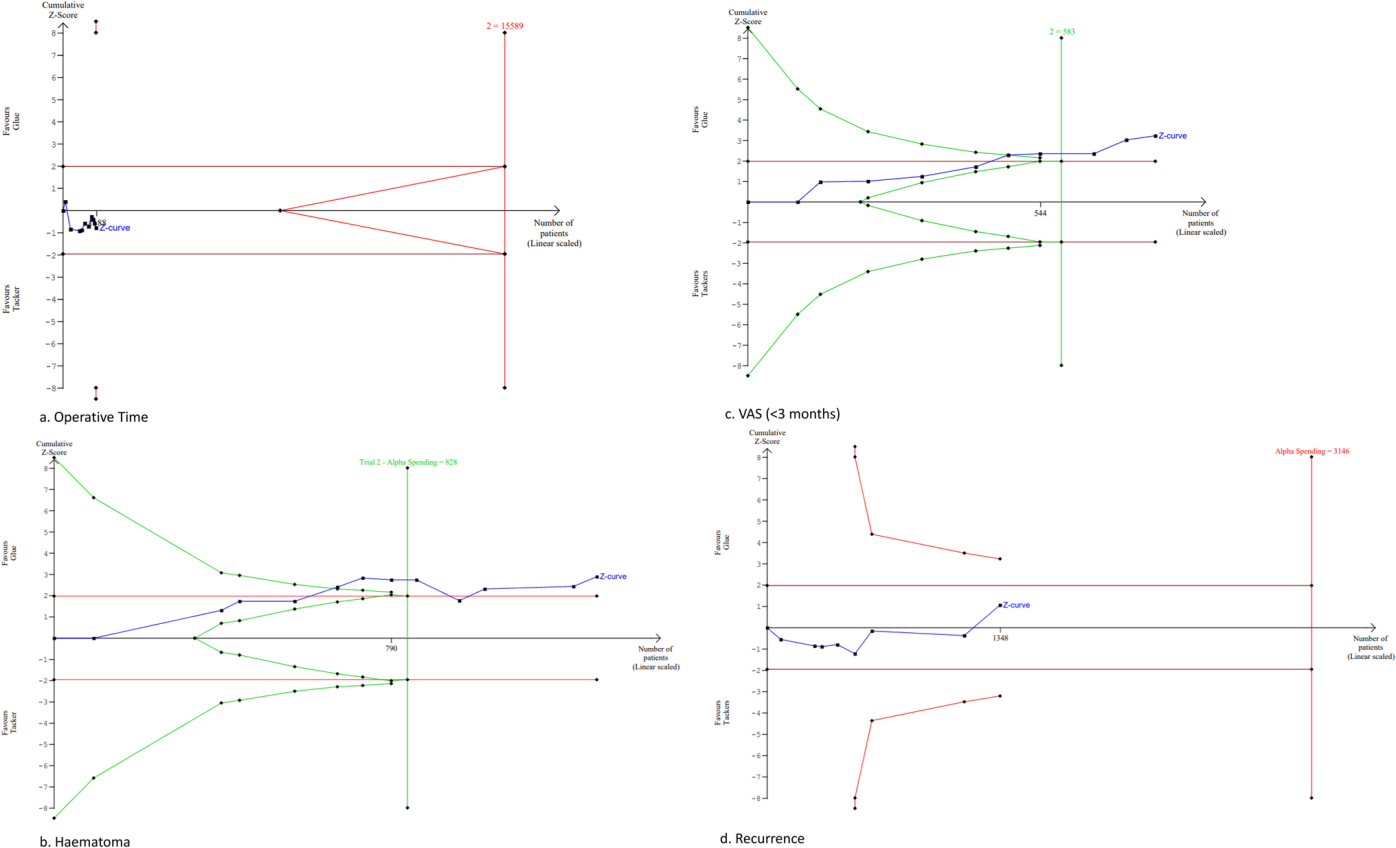
Trial sequential analysis of operative time (**a**), haematoma (**b**), VAS pain after < 3 months (**c**), recurrence (**d**)

The information size for operative time was calculated at 15,589 patients. The information size was not reached for this outcome, and the Z-curve did not cross the conventional, alpha-spending, and futility boundaries; therefore, the meta-analysis was inconclusive, and the risk of type 2 error cannot be excluded ****(Fig. 4a, Fig. 5a).

### Postoperative outcomes

#### Early pain VAS (< 3 months)

Twelve studies reported on postoperative pain VAS scores at < 3 months (n = 1035) [7–14, 16, 18, 19, 27]. Patients who underwent glue fixation reported significantly lower VAS scores before 3 months compared to tacker fixation group [MD (95% CI): − 1.80 (− 2.71 to − 0.89), P < 0.01, I^2^ = 94%] (Fig. 3c).

The information size for VAS less than three months postoperatively was calculated at 583 patients. The Z-curve crossed the conventional boundaries in favour of the glue technique after the information size was reached and the penalised Z value remained greater than 1.96; therefore, the meta-analysis was conclusive, and the risk of type 1 error was minimal (Fig. 5c).

### Length of hospital stay

Seven studies reported data on length of hospital stay (n = 782) [10–13, 17–19]. There was no significant difference between groups [MD (95% CI): − 0.17 (− 0.42 to 0.08), P = 0.18, I^2^ = 65%] (Fig. 4d).

**Fig. 6:**
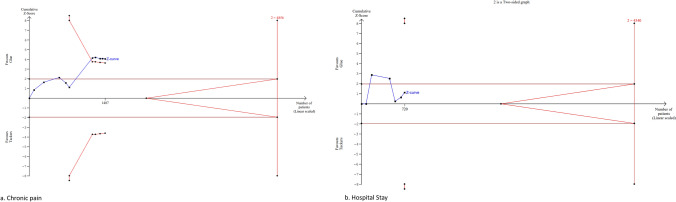
Trial sequential analysis of chronic pain (**a**) and hospital stay (**b**)

The information size for the length of hospital stay postoperatively was calculated at 4540 patients. The information size was not reached for this outcome, and the Z-curve did not cross the conventional, alpha-spending, and futility boundaries; therefore, the meta-analysis was inconclusive, and the risk of type 2 error cannot be excluded (Fig. 6b).

### Return to work/activity

Five studies provided information on a return to work/activity (n = 730) [10–12, 14, 19]. The glue fixation cohort had a significantly shorter time to return to work/activity compared to tacker fixation cohort [MD (95% CI): − 1.92 (− 3.17 to − 0.67), P < 0.01, I^2^ = 93%] (Fig. 3e).

**Fig. 7:**
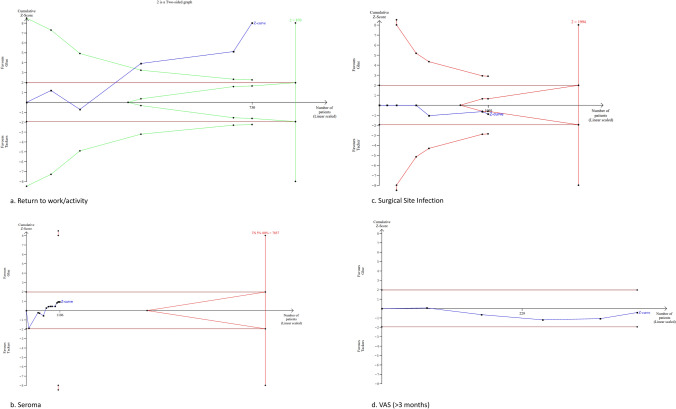
Trial sequential analysis of return to work/activity (**a**), seroma (**b**), surgical site infection (**c**), VAS pain > 3 months (**d**)

The information size for return to work/activity postoperatively was calculated at 870 patients. The Z-curve crossed the conventional boundaries in favour of the glue technique before the information size was reached, and the penalised Z value remained less than 1.96; therefore, the meta-analysis was inconclusive, and the risk of type 1 error cannot be excluded (Fig. 7a).

### Chronic pain VAS (> 3 months post operation)

Seven studies provided data on VAS pain scores > 3 months post-operation (n = 549) [7, 10–12, 14, 18, 19]. There was no significant difference between groups [MD (95% CI): 0.70 (− 0.87 to 2.28), P = 0.38, I^2^ = 98%] (Fig. 4b).

The information size for chronic pain VAS (> 3 months) postoperatively could not be calculated. The Z-curve did not cross the conventional, alpha-spending, and futility boundaries; therefore, the meta-analysis was inconclusive, and the risk of type 2 error cannot be excluded (Fig. 7d).

### Chronic pain incidence

Ten studies reported data regarding post-operative chronic pain (n = 1573) [7, 8, 13–15, 17, 25–28]. The glue cohort had a significantly lower incidence of chronic pain compared to the tacker cohort [MD (95% CI): 0.42 (0.27–0.64), P < 0.01, I^2^ = 28%] (Fig. 4c).

The information size for chronic pain incidence postoperatively was calculated at 4856 patients, which was not reached. The Z-curve crossed the conventional boundaries in favour of the glue technique before the information size was reached, and the penalised Z value remained greater than 1.96; therefore, the risk of type 1 error cannot be excluded (Fig. 6a).

### Haematoma

Twelve studies provided data on postoperative haematoma (n = 1272) [7, 11, 13–19, 24, 25, 27]. Glue fixation was found to significantly reduce postoperative haematoma in comparison to tacker fixation [MD (95% CI): 0.35 (0.17–0.73), P < 0.01, I^2^ = 27%] (Fig. 3b).

The information size for haematoma was calculated at 828 patients. The Z-curve crossed the conventional boundaries in favour of the glue technique after the information size was reached and the penalised Z value remained greater than 1.96; therefore, the meta-analysis was conclusive, and the risk of type 1 error was minimal (Fig. 5b).

### Seroma

Twelve studies reported on the incidence of postoperative seroma (n = 1106) [7, 11, 13–19, 25–27]. There was no significant difference between the two groups in terms of seroma incidence [MD (95% CI): 0.72 (0.33–1.57), P = 0.41, I^2^ = 66%] (Fig. 3f).

The information size for seroma postoperatively was calculated at 7857 patients. The information size was not reached for this outcome, and the Z-curve did not cross the conventional, alpha-spending, and futility boundaries; therefore, the meta-analysis was inconclusive, and the risk of type 2 error cannot be excluded (Fig. 7b).

### Surgical site infection

Six studies reported surgical site infection incidence (n = 1091) [8, 10, 13–15, 28]. There was no significant difference between groups [MD (95% CI): 1.80 (0.52–6.27), P = 0.35, I^2^ = 0%] (Fig. 4a).

The information size for surgical site infection postoperatively was calculated at 1994 patients. The information size was not reached for this outcome, and the Z-curve did not cross the conventional, alpha-spending, and futility boundaries; therefore, the meta-analysis was inconclusive, and the risk of type 2 error cannot be excluded (Fig. 7c).

### Hernia recurrence

Seventeen studies provided data on hernia recurrence (n = 2258) [7–19, 24–26, 28]. The incidence of hernia recurrence was similar between the glue and tacker fixation cohorts [MD (95%): 0.68 (0.34–1.38), P = 0.29, I^2^ = 25%] (Fig. 3d).

The information size for hernia recurrence postoperatively was calculated at 3146 patients, which was not reached. The Z-curve did not cross the conventional, alpha-spending, and futility boundaries; therefore, the meta-analysis was inconclusive, and the risk of type 2 error cannot be excluded (Fig. 5d).

## Discussion

This trial sequential analysis (TSA) compares the effectiveness of glue *versus* tacker mesh fixation during laparoscopic repair of inguinal hernias. This TSA shows that glue fixation reduces early pain scores, and haematoma incidence compared to tacker fixation. The required sample sizes for the TSA of early pain scores and haematoma were reached suggesting that the use of glue provides superior outcomes to tackers in laparoscopic inguinal hernia repair. Tacker use may complicate mesh fixation due to compression of tissue, damaging surrounding lymphatics and vasculature, resulting in post-operative complications such as seroma and haematoma [30, 31]. Fibrin glue applies equivalent adhesive force across the surface of the tissue which may explain the reduction in haematoma formation and early pain.

Interpretation of meta-analyses can result in false positive and negative findings. Trial sequential analysis provides a means of determining significance based on multiple tests, better controlling type I and II errors than traditional meta-analysis. TSA analysis calculates the required number of randomised participants to detect or reject a specific assumed effect. The required information size is defined as the number of participants and events necessary to detect or reject an intervention effect in a meta-analysis. The plotted result of a TSA meta-analysis is displayed on a TSA diagram with four outcome zones including “benefit” and “harm” areas which show statistical significance, and the “not significantly affected” area. The data plotted within the inner wedge area suggests there is strong evidence that further studies will not change the no-effect results [32, 33].

The meta-analysis of 11 randomised controlled trials in this study found no significant difference in operative time between glue and tackers, which is in concordance with the literature [34, 35]. Given cost is a driving factor influencing the use of glue versus tackers, it is interesting Lovisetto et al. found, based on 1 ml of glue usage, that tackers were 70% more expensive; considerable cost savings would be gained from reduced post-operative pain and complications with the use of glue [8].

The recurrence rate for inguinal hernias varies depending on location and type of repair, with 12.3% recurrence over 10 years for open repair [36]. Laparoscopic hernia repair significantly reduces the risk of recurrence compared to open repair (P < 0.01), with a rate at two years of 3.8% [37], to 6.3% over four years [1, 38]. The recurrence rate in this study was 2.0% and 1.8% for glue and tackers respectively, at two years. We have importantly shown that there is no difference in risk of recurrence with glue and tackers, as this would negate any benefit found regarding recovery. Previous meta-analyses have shown similarly that there is no difference in recurrence rate when comparing glue and tackers [34, 39].

The limitations of this study include the lack of detail regarding the method of randomisation in five studies, increasing the potential risk of bias in patient selection. In addition, six studies used alternative pain scales to VAS scores or provided time points not consistent with this study's collection of less than three months and greater than three months, which increases the heterogeneity of results. Chronic pain incidence was defined differently between studies and was followed up over different time frames making the comparison less accurate. TSA of return to activity and chronic pain incidence found the cumulative z-line crossed the boundary for effect but did not reach the required sample size. This indicates that although mesh fixation with glue significantly reduces the return to activity time and chronic pain incidence, further studies are required to obtain definitive evidence on the benefit of glue in these aspects. No significant difference was found in seroma formation due to the sample size for seroma not reaching the minimum threshold. Studies did not consistently report size of hernia and the type, size and weight of mesh used which may contribute to post-operative pain.

In conclusion, the trial-sequential analysis showed that glue fixation reduced early pain scores and haematoma formation; the remaining tested variables did not meet the required minimum information size for TSA with further randomised control trial evidence needed. The meta-analysis of these variables showed that glue fixation may reduce the time for a patient to return to normal activity, with less pain and reduced complications.
